# The power of online panel paradata to predict unit nonresponse and voluntary attrition in a longitudinal design

**DOI:** 10.1007/s11135-022-01385-x

**Published:** 2022-04-25

**Authors:** Sebastian Kocar, Nicholas Biddle

**Affiliations:** 1 Institute for Social Change, University of Tasmania, Hobart, Australia; 2 Centre for Social Research and Methods, The Australian National University, Canberra, Australia

**Keywords:** Online panel paradata, Panel voluntary attrition, Unit nonresponse, Random-effect logit model, Prediction modeling

## Abstract

The objective of this study is to identify factors affecting participation rates, i.e., nonresponse and voluntary attrition rates, and their predictive power in a probability-based online panel. Participation for this panel had already been investigated in the literature according to the socio-demographic and socio-psychological characteristics of respondents and different types of paradata, such as device type or questionnaire navigation, had also been explored. In this study, the predictive power of online panel participation paradata was instead evaluated, which was expected (at least in theory) to offer even more complex insight into respondents’ behavior over time. This kind of paradata would also enable the derivation of longitudinal variables measuring respondents’ panel activity, such as survey outcome rates and consecutive waves with a particular survey outcome prior to a wave (e.g., response, noncontact, refusal), and could also be used in models controlling for unobserved heterogeneity. Using the Life in Australia™ participation data for all recruited members for the first 30 waves, multiple linear, binary logistic and panel random-effect logit regression analyses were carried out to assess socio-demographic and online panel paradata predictors of nonresponse and attrition that were available and contributed to the accuracy of prediction and the best statistical modeling. The proposed approach with the derived paradata predictors and random-effect logistic regression proved to be reasonably accurate for predicting nonresponse—with just 15 waves of online panel paradata (even without sociodemographics) and logit random-effect modeling almost four out of five nonrespondents could be correctly identified in the subsequent wave.

## Introduction

Panels as online survey methods are now routinely used for collecting data and have been increasing in number. The online panel survey mode has introduced new sources of survey errors, even compared to traditional longitudinal surveys/non-online panels, such as birth cohort studies or life-cycle studies. There are at least two important elements related to these survey errors, which are specific to longitudinal surveys and panels collecting cross-sectional survey data: panel conditioning and attrition. In online panel studies, attrition is predominantly considered as permanent nonresponse from a particular data collection wave onwards (Kocar 2020). Besides attrition, unit nonresponse/survey non-completion is another potential source of representation bias (Groves et al. 2009), although clearly not specific to online panel surveys.^1^ While response rates alone are not a reliable indication of error, and it has been reported that the association between response rate and bias is weak at best (Groves and Peytcheva 2008), a high unit nonresponse typically signals a higher likelihood of nonresponse bias (Baker et al. 2010). Further, respondents who opt-out should at some point be replaced on the panel with new respondents to preserve adequate sample size—particularly for certain population sub-groups—which increases the costs of panel management and data collection (Kruse et al. 2009).

Online panels should be considered a form of hybrid between traditional longitudinal studies and web surveys since they predominantly use the online survey mode for collecting data from panel members but track individuals over time, even though longitudinal outcomes are not always the focus of the data collection. That often includes collection of paradata specific to online panels and storing the entire history of each member’s panel behavior. Since this class of paradata have been a less explored topic (Callegaro 2013) and psychological theory explains that past behavior predicts future behavior fairly well (e.g., see Ouellette & Wood 1998), in this study we firstly review differential nonresponse and attrition. We then investigate the predictive power of online panel paradata to mitigate the problem of nonparticipation in probability-based online panel research. The main aim of this study is to explore the added value of this type of paradata in identifying future nonparticipation with complex statistical modeling. Due to the panel nature of this the data, we can also establish the value of panel data analysis controlling for unobserved heterogeneity in predicting nonresponse and attrition. Accurate identification of future nonparticipation could ultimately lead to a reduction in nonresponse error, as defined in Total Survey Error framework (Groves et al. 2009), if nonparticipants were successfully treated to prevent them from not completing future panel questionnaires [e.g., with tailored incentives (Lugtig 2014)].

In this article, we first present a literature review of the role of paradata in online panels, including how they have been used analytically to study panel participation. Second, we present our methodological approaches with an emphasis on data analysis, statistical modeling, and covariates that we have derived from online panel paradata. Third, we present the results on the predictive power of socio-demographics and panel paradata in different statistical models and provide examples of how the findings from this study could be applied in practice. We conclude with a discussion about the relevance of this investigation and outline several practical recommendations and future research opportunities.

## Literature review

### Unit nonresponse and attrition in online panels

Unit nonresponse or survey non-completion, including non-contact, refusal, or break-off,^2^ is a source of representation bias in online panel surveys (and surveys in general), especially with respect to the demographic or attitudinal characteristics of panel members (Groves et al. 2009). Moreover, we can distinguish between two types of attrition in panel studies: forced and normal. While forced attrition is managed by the data collector and occurs systematically at the end of eligibility, normal attrition is not managed and is a form of nonresponse; it occurs when panel members do not reach the end of their eligibility and leave the panel earlier for a variety of reasons, such as opting out, not participating fully, or falsifying interviews (Baker et al. 2010). In this study, we use a classification by Callegaro and DiSogra (2008), who introduced slightly different online panel attrition outcomes: voluntary attrition, involuntary attrition, and mortality, with the focus on voluntary (or opt-out) attrition.

Both unit nonresponse and attrition may be considered sources of non-random survey errors (Cheng et al. 2016) in the case of differential nonparticipation (i.e., nonresponse and voluntary attrition). It has been previously reported that voluntary attrition not only decreases the online panel sample size; selective attrition may introduce additional biases on top of that due to recruitment (Lugtig 2014). Once both sources of nonparticipation are combined, the representation bias tend to increase, and nonignorable nonresponse can be the reason why even refreshment samples cannot fully correct for attrition bias (Schifeling et al. 2015).

In web surveys, response rates are significantly influenced by numerous factors, such as the questionnaire topic, length, sequencing, formatting, sampling method, whether participation is by invitation or not, pre-notification, and reminders (Fan and Yan 2010), as well as by socio-demographic characteristics such as age, education, income, race, and ethnicity (e.g., Callegaro et al. 2015; Couper et al. 2007; Tourangeau et al. 2013), and computer literacy and internet use (e.g., Callegaro et al. 2015; Tourangeau et al. 2013). In longitudinal and online panel studies, there are several predictors of attrition and response and some of these are specific to the panel format—Watson and Wooden (2009) concluded that it could not be assumed that experience with nonresponse in cross-sectional surveys is always relevant for predicting response and attrition in longitudinal surveys, and there is a large random component to survey nonresponse. Besides demographic and socio-economic characteristics such as gender, age, education, race, household composition and size, urbanicity, home ownership, and country of birth (e.g., Kruse et al. 2009; Lugtig et al. 2014; Rübsamen et al. 2017; Uhrig 2008; Watson and Wooden 2009), respondents’ personalities could be a source of differential nonresponse and attrition (e.g., Cheng et al. 2016; Hansson et al. 2018; Lugtig 2014).

There are also observable characteristics in the interview process that are predictive of unit nonresponse in a panel study. For example, respondents’ perception of the survey in the preceding longitudinal study wave might influence cooperation in future waves (Watson and Wooden 2009), and item nonresponse can be predictive of future unit nonresponse (Loosveldt and Billiet 2002). While incentives are commonly used in longitudinal and online panel research (e.g., Castiglioni et al. 2008; Kocar and Kaczmirek 2021), an individual’s initial motivation to participate in a study might also be related to attrition probability, whereby those motivated strictly by monetary incentives have a higher probability of attrition (Frankel and Hillygus 2014). Lastly, for panel management purposes, panellists can also be classified according to response type and attrition group, such as “stayers”, “late-comers”, “fast attritors”, and “lurkers”, to help understand their future participation; while stayers participate in almost all waves, lurkers are infrequent respondents, attritors opt-out of the panel at some point, and fast attritors leave even earlier (Lugtig 2014).

### Paradata and their use in online panels

Paradata in surveys can be defined as additional data captured during the process of generating survey statistics and can be collected at different stages with different levels of detail (Kreuter 2013). Hence, different classifications, types, and possible applications of paradata exist. In web surveys, paradata may be categorized into (1) device-type paradata (e.g., device, browser, and operating system (OS) used), and (2) questionnaire navigation paradata (e.g., mouse clicks, order of answering, last question answered before breaking off, and time spent per question). In addition to those for cross-sectional web surveys, there is a separate class of paradata—online panel paradata, which includes survey invitations received, surveys completed, attrition, and survey topics (Callegaro 2013). Web survey paradata can be collected in different phases: prior survey phase, recruitment phase, access phase, and response phase (McClain et al. 2019), and can be used for examining total survey errors (McClain et al. 2019; Olson and Parkhurst 2013), nonresponse (Lynn 2017) and panel attrition (Lugtig and Blom 2018; Roßmann and Gummer 2016), or for calculating propensity score weights adjusting for attrition (Roßmann and Gummer 2016). Lugtig and Blom (2018) concluded that paradata-identified behavior largely predicts nonresponse and Roßmann and Gummer (2016) reported an improvement in the fit of the nonresponse model after adding respondents’ participation history, while both studies used a limited number of variables from paradata specific to online panels (e.g., participation in the previous wave). However, as Callegaro (2013) concluded, paradata for online panels are still a little explored topic, especially in a longitudinal design which takes advantage of the ability to derive longitudinal types of predictors. Also, longitudinal/panel data analysis methods controlling for unobserved heterogeneity can be used.

### Statistical methods to study panel participation with panel paradata

To study panel participation, “static” statistical methods, such as survival analysis (Kruse et al. 2009), logistic regression (Castiglioni et al. 2008; Roßmann and Gummer 2016), multiple linear regression (Cheng et al. 2016), classification and regression trees (Lugtig and Blom 2018), and other tree-based machine learning methods such as boosting methods (Kern et al. 2019) have generally been used in previous studies. On the other hand, there are several advantages of analyzing paradata in a panel form using dynamic panel data modeling techniques. Analyzing panel data offers more accurate inference of panel parameters, greater capacity to capture complex behavior (including controlling the impact of omitted variables and generating more accurate predictions) and simplifying computation and statistical inference while involving at least two dimensions: a cross-sectional one and a time-series one (Hsiao 2007, pp. 3–6). In case of binary outcome variables (such as survey response in a wave, 1 = yes, 0 = no), binary logistic panel data analysis should be used instead of more traditional linear panel data models (see Bartolucci and Nigro 2010). The challenge of any panel data analysis to obtain valid inference on structural parameters is to control the impact of unobserved heterogeneity, which effects can either be assumed as random variables (random-effect model), as fixed parameters (fixed-effect model), or both (mixed-effect model) (Hsiao 2007, p. 8). An alternative is using pooled data analysis, which is fundamentally applying classical regression (e.g., linear or logit) to pooled data. While this type of regression obtains minimum variance estimates of covariates under certain conditions, fixed-effect and random-effect models would often minimize variance better while accommodating a greater variety of covariates and sample sizes (Ward and Leigh 1993).

### Outline of the study

This study investigates the differential nonparticipation in probability-based online panels and the power of online panel paradata predictors of nonparticipation rates, i.e., nonresponse and voluntary attrition rates. In contrast to similar research in the field, longitudinal panel participation data, i.e., survey outcome statuses, are explored in detail. The longitudinal nature of paradata enable the derivation of a number variables measuring panel response behavior over time. Panel data analysis will be carried out, which include the dimension of time in the models to improve the accuracy of the predictions. This study aims to answer the following research questions (RQs):

1. What is the extent of differential nonresponse and differential voluntary attrition in probability-based online panel surveys?

The theory on nonresponse in longitudinal and online panel studies suggests that there are a number of socio-demographic characteristics associated with nonparticipation (e.g., Kruse et al. 2009; Lugtig et al. 2014; Rübsamen et al. 2017; Uhrig 2008; Watson and Wooden 2009). By answering this question, we will also determine if the available socio-demographic predictors should be included in regression models to improve the accuracy of prediction with online panel paradata (this will also contribute to addressing RQ2).

RQ2: What is the predictive power of online panel paradata with or without socio-demographics?

Assuming we identify some level of differential nonresponse in online panels, we will compare the predictive power of online panel paradata with and without socio-demographics using logit regression modeling (see Castiglioni et al. 2008; Roßmann and Gummer 2016). This comparative approach is similar to behavioral research in psychology where personality traits and past behavior as predictors of future behavior are compared (e.g., Harris et al. 2016). We will use online panel paradata to derive various predictors of future panel participation.

RQ3: To what extent do random-effect models as dynamic logistic regression models improve the accuracy of prediction in comparison to pooled “static” regression models, if at all?

We will also show if using the advantages of the panel structure of the data, which is to create dynamic regression models, can increase the accuracy of prediction of participation in probability-based panels in contrast to pooled estimation with panel data as reported in the literature (e.g., Castiglioni et al. 2008; Cheng et al. 2016; Kruse et al. 2009; Roßmann and Gummer 2016). Generally speaking, fixed-effect within-person regression could be more accurate in identifying behavioral indicators of nonresponse and their magnitude (if explanatory variables are correlated with the error term), but it would not be possible to use its model coefficients to calculate the predicted probabilities for each respondent.

RQ4: How many waves of online panel participation data are needed to predict nonparticipation with desirable accuracy?

Since our time-series is much longer in comparison to the studies carried out by Lugtig and Blom (2018) and Roßmann and Gummer (2016), we will provide insight into how much data are required for fairly accurate prediction using the most accurate model (pooled or random-effect, with or without socio-demographics).

RQ5: How do we determine the right balance between “costs” and “benefits” when identifying nonrespondents for further treatment?

Identifying potential nonrespondents itself would have little value for an online panel organization without following with some form of treatment to increase response and decrease attrition (e.g., Lugtig 2014). We will show how identification as the first step in improving participation becomes inefficient and cost-ineffective at some level and discuss practical solutions to that.

## Methods

### Data

The dataset used in this research was all members of the Life in Australia™, the only mixed-mode probability-based online panel in Australia. The Life in Australia™ dataset used in this study did not consist of substantive survey data, but of panel response, attrition, incentives, and other characteristics of the panel members for waves 1–30 (data collection period: December 2016 and August 2019). There was a substantial panel refresh after this period, which, in addition to the 2019/20 Black Summer bushfire season and the COVID-19 pandemic, introduced the strong potential for a structural break in the dataset. This data collection period is therefore well suited to a focused research program.

It was possible to use the dataset to study survey participation, including nonresponse and panel attrition, and included information for 3322 panel members whose demographic information had been collected at the end of 2016 (Kaczmirek et al. 2019). The relatively small top-up sample from May 2018 is not included in this study. For each of the 30 waves of subsequent data collection, the dataset included all relevant information about the activity of panel members. If a panel member became inactive (excluding vacations or public holidays) due to voluntary (panel opt-out) or involuntary (retired) attrition, or due to mortality (death), participation data were no longer collected for that respondent from the successive wave as attrited units are no longer relevant for analysis (cannot rejoin and re-attrite, hence no variability in response). These missing data make the panel an unbalanced panel in panel data analysis.

### Population and sampling

The population in this research was defined as “Australian residents aged 18 years or older”. The recruitment rate for the establishment of the Life in Australia™ panel was 21.1% and the profile rate was 77.7%. For the recruitment process, a dual-frame random digit dialing (RDD) sample design was employed, with a 40:60 (pilot) and 30:70 (the main recruitment effort) split between landline and cell phone sample frames. The offline population, so-called offliners, completed surveys by telephone (Kaczmirek et al. 2019). All members of the sample were invited to participate in the majority of surveys between December 2016 and August 2019, except for waves 5, 8, 13, and 20. All variable values for all units were, nevertheless, included in the analysis, since the increased time gap between survey invitations could well prove to be one of the predictors of survey participation.

### Data analysis, statistical models, and derived covariates

To analyze the data and to answer the research questions, multivariate statistical analysis was used, including panel data analysis. These models were created to study nonresponse and voluntary attrition (as the outcome variables) using paradata and not for substantive analysis using substantive survey items. Nonresponse, which was predicted using the individual-level paradata and socio-demographic characteristics of the online panellists, was defined as any survey non-completion outcome. Voluntary attrition, explored using individual-level paradata and socio-demographic characteristics, was a binary outcome variable in these models, with “0” representing non-attrition (remaining in the panel) and “1” representing panel “opt-out” attrition (voluntarily leaving the panel).

In addition to multiple linear regression analysis and binary logistic regression (aggregated participation variables, RQ1), this study used logistic regression analysis for the binary panel data in the main models (RQ2-RQ5). The added value of panel data analysis would be consideration of the longitudinal dimensions of survey participation. We will use dynamic logit models, which were previously adopted to allow for the use of binary panel data, to disentangle true state dependence from the propensity to experience outcomes in all periods. For subject i at occasion t, the basic assumption (i = 1, …, n, t = 1, …, T) is presented in Eq. 1 (from Bartolucci and Nigro (2010)):

1 $$\mathrm{log}\frac{p\left({y}_{it}=1|{\alpha }_{i},{x}_{it}\right)}{p\left({y}_{it}=0|{\alpha }_{i},{x}_{it}\right)}={\alpha }_{i}+{x}_{it}^{\mathrm{^{\prime}}}\upbeta +{y}_{i,t-1}\gamma$$

where n is the sample size, T is the total number of occasions, ${y}_{i,t}$ is the binary response variable, x is a vector of exogenous covariates, ${\alpha }_{i}$ are individual-specific parameters for the unobserved heterogeneity and β and γ are structural parameters. The selected longitudinal or panel data in this study consisted of repeated observations of the same units at different points in time, enabling control for unobserved heterogeneity.^3^

Table 1 presents all multivariate models used in this study to address the research questions. We used a step-by-step approach in identifying the most suitable prediction models by firstly determining the value of socio-demographics as predictors, and secondly establishing the value of dynamic models controlling for unobserved heterogeneity (consistent with the order of our research questions). After determining that the accuracy of identifying voluntary attritors was very low, we decided to exclusively focus on nonresponse in the remaining analyses.

**Table 1:** Statistical models used in this study (by research question)

Research question	Model	Outcome variable	Predictors
RQ1 *(differential nonresponse and attrition)*	Multiple linear regression model	Individual survey completion rate^a^	Socio-demographics^c^
	Binary logistic regression model	Individual voluntary attrition at any point in time^b^	
RQ2 *(predictor choice)*	Binary (pooled) logistic regression model	Voluntary attrition in a particular wave^d^	Online panel paradata variables (with and without socio-demographics^c^)
	Binary (pooled) logistic regression model	Nonresponse in a particular wave^e^	
RQ3 *(modeling choice)*	Binary (pooled) logistic regression models	Nonresponse in a particular wave^e^	Online panel paradata variables and socio-demographics^c^
	Random-effect logit models	Nonresponse in a particular wave^e^	
RQ4, RQ5 *(length of time series and cost–benefit analysis)*	Random-effect logit models	Nonresponse in a particular wave^e^	Online panel paradata variables and socio-demographics^c^

Fixed-effect models are added as a sensitivity analysis; see Tables 5 and 6 in the Appendix.

^a^Calculated as: (number of all completed questionnaires / all panel waves invited to)

^b^A binary variable with values: 1 = opted-out in the first 30 waves, 0 = still a panel member after 30 waves

^c^Gender, education, capital city in state, born in Australia, only English spoken at home, indigenous status, other healthcare card, carer status, population (online, offline), age group, Socio-Economic Indexes for Areas (we performed multiple imputations for missing socio-demographic data in Stata)

^d^A binary variable with values: 1 = opted-out in wave_n_, 0 = remained in the panel after wave_n_

^e^A binary variable with values: 1 = nonresponse in wave_n_, 0 = survey completion in wave_n_

The derived variables as exogenous covariates/predictors of panel participation in pooled and random-effect models were predominantly based on the AAPOR categorization of the survey outcome rates (see The American Association for Public Opinion Research 2016). The predictors derived from each panellist’s questionnaire completion history (recorded by online panel paradata) using different calculation and derivation approaches are presented in Table 2 below.

**Table 2:** Derived variables as exogenous covariates/predictors of panel participation

Predictor	Calculation
Participation rate *(prior to wave*_*n*_*)*	$\frac{\mathrm{number\, of \,all \,completed \,questionnaires\, by\, }{\mathrm{wave}}_{\mathrm{n}} }{\mathrm{total\, number \,of \,waves\, by\, }{\mathrm{wave}}_{\mathrm{n}}}$
Non-contact rate *(prior to wave*_*n*_*)*	$\frac{\mathrm{number\, of \,noncontacts \,by\, }{\mathrm{wave}}_{\mathrm{n}} }{\mathrm{number \,of \,all \,panel \,waves \,invited \,to \,by\, }{\mathrm{wave}}_{\mathrm{n}}}$
Refusal rate *(prior to wave*_*n*_*)*	$\frac{\mathrm{number\, of \,all \,refusals \,by\, }{\mathrm{wave}}_{\mathrm{n}} }{\mathrm{number \,of \,all \,panel \,waves \,invited \,to \,by\, }{\mathrm{wave}}_{\mathrm{n}}}$
Non-refusal rate *(prior to wave*_*n*_*)*	$\frac{\mathrm{number\, of \,all \,non\,}-\mathrm{refusals\, by\, }{\mathrm{wave}}_{\mathrm{n}} }{\mathrm{number\, of \,all \,panel \,waves \,invited \,to \,by\, }{\mathrm{wave}}_{\mathrm{n}}}$
Charity rate *(prior to wave*_*n*_*)*^a^	$\frac{\mathrm{number \,of \,donations \,to \,charity \,by\, }{\mathrm{wave}}_{\mathrm{n}} }{\mathrm{number \,of \,all \,panel \,waves \,with \,completed \,questionnaires \,by\, }{\mathrm{wave}}_{\mathrm{n}}}$
Consecutive participation *(prior to wave*_*n*_*)*	Consecutive waves prior to wave_n_ with completed questionnaires (invited or not)
Consecutive response *(prior to wave*_*n*_*)*	Consecutive waves prior to wave_n_ with completed questionnaires (waves invited to only)
Consecutive non-contact *(prior to wave*_*n*_*)*	Consecutive waves prior to wave_n_ with noncontact survey outcome (waves invited to only)
Consecutive refusal *(prior to wave*_*n*_*)*	Consecutive waves prior to wave_n_ with refusal survey outcome (waves invited to only)
Consecutive non-refusal *(prior to wave*_*n*_*)*	Consecutive waves prior to wave_n_ with non-refusal survey outcome (waves invited to only)
Consecutive charity donations *(prior to wave*_*n*_*)*	Consecutive waves prior to wave_n_ with donations to charities (waves with completed questionnaires only)
Change from interview to other *(prior to wave*_*n*_*)*	Interview survey outcome in wave_n-2_ and nonresponse (non-contact, refusal, or non-refusal) in wave_n-1_ (waves invited to only)
Change from other to refusal *(prior to wave*_*n*_*)*	Interview, non-contact, or non-refusal survey outcome in wave_n-2_ and refusal in wave_n-1_ (waves invited to only)

^a^Charity rate is a special type of rate and is not one of standard survey outcome rates. Yet, it is associated with motivation to participate in online panel surveys and could be treated as a type of panel behavior measured with online panel paradata. The same can be said for consecutive charity donations

For each type of survey outcome, the rates prior to a wave of data collection (and consecutive outcomes) were calculated for each respondent in the panel. For example, the participation rate prior to wave 6 was the total participation rate for waves 1, 2, 3, 4, and 5 for that panellist.^4^ While initially derived, response rate covariate was later excluded since it was highly correlated with the participation rate and was a linear combination of the other survey outcome rates. Also, the difference between consecutive participation and consecutive response was in waves that a panellist was not invited to—non-invitation was counted as nonparticipation, but not as nonresponse. Changes between survey outcomes were possible to calculate from wave 3 on, since two consecutive waves of data were required to identify changes in a panellist’s participation behavior prior to a wave. Changes from interview to other outcomes (including non-contact, non-refusal, physical or mental inability/incompetence, but excluding refusal) in consecutive waves were the less considerable changes of survey response outcomes, while any other survey outcome (including interview) to refusal should be considered as a more severe change and potentially a better predictor of future nonresponse or voluntary attrition.

### Prediction of panel participation

The accuracy of prediction was calculated following the next steps:

- Step 1: using pooled and random-effect logit regression modeling with online panel paradata predictors (and socio-demographics), we calculated probabilities of questionnaire non-completion in the subsequent wave for each panellist, which was a continuous variable between 0 (the lowest chance of non-completion) and 1 (the highest chance of non-completion); we used data for waves 1–3 to predict nonresponse in wave 4, data for waves 1–4 to predict nonresponse in wave 5, and data for waves 1–29 to predict nonresponse in wave 30;
- Step 2: having information on actual response (invited panellists, nonrespondents) in the subsequent wave, we selected the same number of panellists with the highest probabilities of questionnaire non-completion from Step 1 (e.g., in wave 4, there were 2424 actual respondents and 566 actual nonrespondents, and so we assigned nonresponse to 566 panellists with the highest probabilities of non-completion based on wave 1–3 data);
- Step 3: we compared (1) actual respondents and nonrespondents with (2) predicted respondents and nonrespondents and calculated prediction efficiency for a particular wave.

To compare the prediction power of online panel paradata and socio-demographics, pooled regression models and random-effect models, we presented two key statistics: accuracy and recall. Accuracy was used as a metric for correct identification of both respondents and nonrespondents in the subsequent wave, and recall, calculated as true positives divided by all actual positives, was used as a metric for correct identification of nonrespondents only. Since the propensity for survey completion was about four times as high as nonresponse in Life in Australia™, accuracy of any model (or even random selection) should naturally be higher than recall. As we worked with full online panel paradata including response numbers for all 30 waves of data collection, we did not need to estimate nonresponse in the subsequent waves to determine the target number of nonrespondents identified with our prediction models, something that would need to be done in real-life situations. This way, precision as the third metric typically reported in data science to evaluate algorithms, is equal to recall and thus does not need to be reported. We present results for waves 4–30 since we needed at least three waves of data to derive certain behavioral predictors and to avoid multicollinearity.

## Results

In this section, we present all results and address the research questions (each subsection addresses a separate research question). For basic descriptive statistics (bivariate analysis), see Table 4 in the Appendix. Of all Life in Australia™ panellists recruited in 2016 (n = 3322), only those who were once active (i.e., responded in at least one wave out of 30) were included (n = 2990). The groups with the lowest survey completion rates were the youngest panellists, respondents who spoke a language other than English at home and those who self-identified as Indigenous. On the other hand, the groups most likely to opt-out of the panel were the least educated and those completing the surveys offline, and voluntary attrition generally increased with age. At the same time, the association between the survey response rate and voluntary attrition indicates that attritors respond with a lower propensity than non-attritors, even before opting out of the panel. The relationship between socio-demographics and nonresponse, as well as socio-demographics and voluntary attrition will be further investigated with regression analysis to address RQ1.

### Socio-demographic predictors of panel nonresponse and attrition

To extend the descriptive analysis, the first multiple linear regression model demonstrated the effects of the characteristics of the online panel respondents (as the independent variables) on the nonresponse rate (as the continuous dependent variable). The second logistic regression model demonstrated the effects of the same characteristics on voluntary attrition (as the binary dependent variable). The evidence from Table 3 helped answer the first research question regarding differential nonresponse and differential attrition (RQ1).

**Table 3:** Multiple linear regression (survey completion rates) and logistic regression (voluntary attrition) results, socio-demographic predictors, waves 1–30, 2872 persons

	Survey completion rate		Voluntary attrition	
	Coef.	p value	Coef.	p value
Gender				
Female	0		0	
Male	− 1.70	0.113	− 0.03	0.811
Education				
Bachelor or higher	0		0	
Certificate/diploma/trade	− 6.96	< 0.001**	0.14	0.310
Year 12 or equivalent	− 3.86	0.036*	− 0.05	0.814
Year 11 or less	− 9.29	< 0.001**	0.37	0.029*
Capital city in state				
No	0		0	
Yes	1.43	0.263	0.05	0.718
Born in Australia				
No	0		0	
Yes	1.95	0.141	− 0.08	0.571
Only English spoken at home				
No	0		0	
Yes	6.32	< 0.001**	0.19	0.341
Indigenous status				
No	0		0	
Yes	− 3.38	0.362	− 0.44	0.358
Other healthcare card				
No	0		0	
Yes	− 0.36	0.779	− 0.28	0.041*
Carer status				
No	0		0	
Yes	4.27	0.002**	− 0.59	< 0.001**
Population				
Offline	0		0	
Online	8.96	< 0.001**	− 0.57	< 0.001**
SEIFA				
Quartile 1	0.12	0.947	0.06	0.791
Quartile 2	− 1.15	0.513	0.30	0.110
Quartile 3	0.00		0	
Quartile 4	− 1.62	0.337	0.43	0.019*
Quartile 5	− 3.83	0.023*	0.32	0.086
Age	0.45	< 0.001**	0.02	< 0.001**
Constant	43.99	< 0.001**	− 2.84	< 0.001**
Adjusted R-squared	0.085			
Pseudo R-squared			0.044	

*Coef* model regression coefficient

* Significant at the 0.05 level

** Significant at the 0.01 level

The results of the regression analyses showed that the overall individual response rate for all waves was positively associated with the highest education (the coefficients (coef.) for certificate/diploma/trade and Year 12 or equivalent, Year 11 and lower were all below -3, at p < 0.05), only English spoken at home (coef. 6.32 and p < 0.001), carer status (coef. 4.27 and p < 0.01), and being older (age, a continuous variable, coef. 0.45 and p < 0.001). The online population tended to produce a higher response rate than the offline respondents (coef. 8.96 and p < 0.001) and the Socio-Economic Indexes for Areas (SEIFA) Quartile 5 group tended to respond less frequently (coef. − 3.83 and p < 0.05), ceteris paribus. The adjusted R-Squared value equaled 0.085, meaning that the model explained 8.5% of the variability in the response data. While that indicates that differential nonresponse is present, it does not seem to be severe.

The effects of socio-demographic predictors on the binary dependent variable in the logit regression model voluntary attritor can also be seen in Table 3. The results showed that panel opt-out attrition in the first 30 waves (0 = no, 1 = yes) was positively associated with the lowest education level (Year 11 or less, coef. 0.37 and p < 0.05) and age (coef. 0.02 and p < 0.01), and negatively associated with holding other healthcare card (coef. − 0.28 and p < 0.05), carer status (coef. − 0.59 and p < 0.01), and online population status (coef. − 0.57 and p < 0.01).

In the next paragraphs, we will focus on prediction of nonparticipation by combining socio-demographics data with online panel paradata.

### Online panel paradata predictors of panel nonresponse and voluntary attrition

To extend the analysis in Sect. 4.1 and to answer RQ2 (predictor choice), we used pooled logit regression analysis with non-aggregated data. We investigated how accurately nonrespondents and voluntary attritors could be identified using their previous panel participation behavior (1) with socio-demographic predictors and (2) without socio-demographic predictors.

Firstly, we must emphasize that the accuracy of identifying voluntary attritors was fairly low, i.e., recall was equal to less than 20% in any models we constructed, with or without socio-demographics, pooled or random-effect modeling (for more information see Table 6 with regression results in the Appendix), and no matter how many future waves were investigated. We concluded that predicting nonrespondents (and treating them) should offer better results in dealing with potential voluntary attrition, and the remaining analyses are focused on the prediction of nonresponse.

**Fig. 1:**
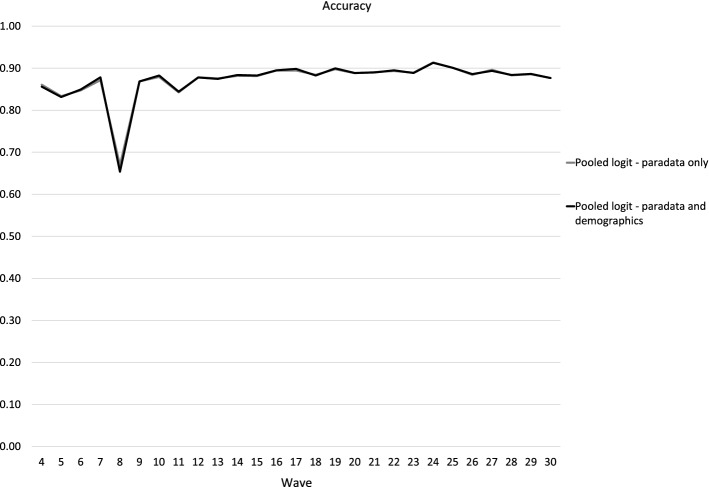
Predictive power for response and nonresponse combined, paradata prediction with and without socio-demographics, waves 4–30 (Accuracy)

The accuracy curves in Fig. 1 show the total accuracy of identification of both respondents and nonrespondents in a certain wave, with two different ranges of predictors. We observed very little to no differences between models with or without socio-demographic predictors. Using the original data, models with online panel paradata predictors were more accurate since there were about 4% of panellists with incomplete socio-demographic data, and this missingness was also associated with a lower propensity to respond in a particular wave. We corrected this problem with multiple imputations, resulting in an improved accuracy of models including socio-demographic variables. After imputation, there was almost no difference.

**Fig. 2:**
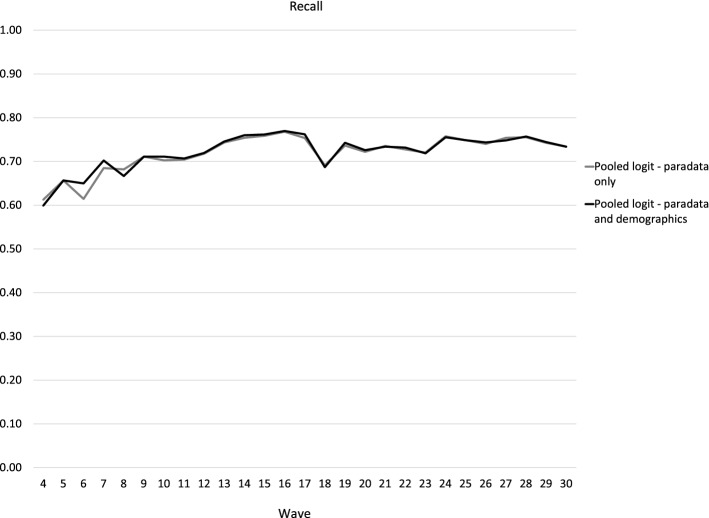
Predictive power for nonresponse, paradata prediction with and without socio-demographics, waves 4–30 (Recall)

The recall curves in Fig. 2 show the accuracy of identification of nonrespondents in a certain wave. We again cannot observe substantial differences between models with different ranges of predictors (especially not after wave 10), and multiple imputations for missing socio-demographic information improved efficiency by about 3% in the models including socio-demographic predictors. On average, socio-demographic covariates add very little predictive power to online panel paradata predictors.^5^

### Modeling panel nonresponse

**Fig. 3:**
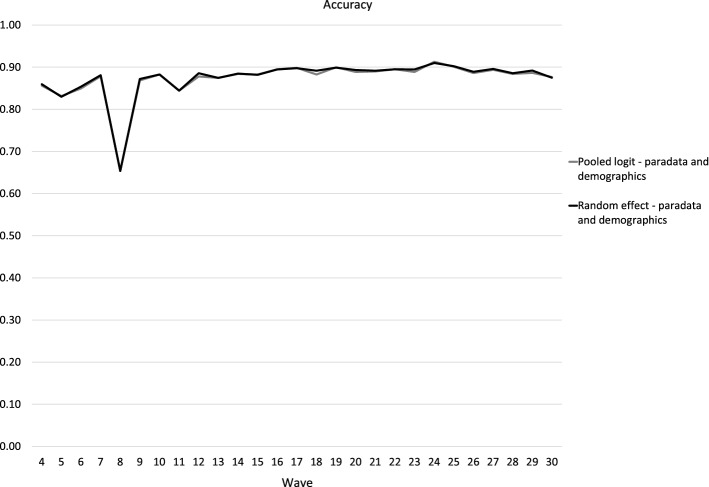
Predictive power for nonresponse, pooled logit and random-effect logit regressions, waves 4–30 (Accuracy)

**Fig. 4:**
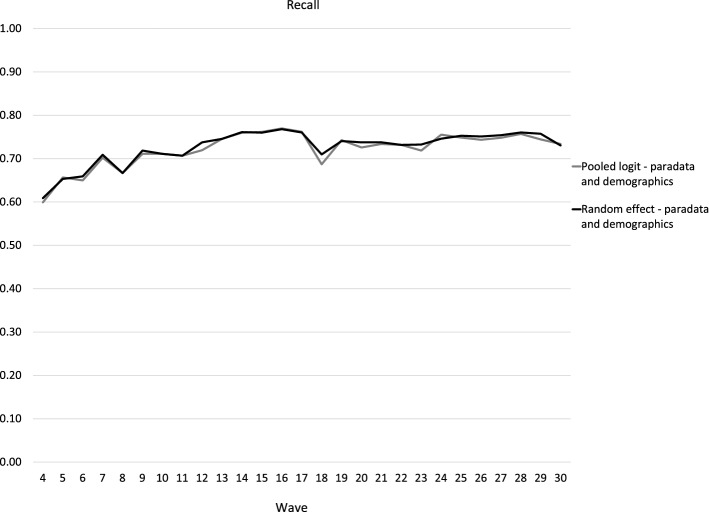
Predictive power for response and nonresponse combined, pooled logit and random-effect logit regressions, waves 4–30 (Recall)

To answer RQ3 (modeling choice), we investigated how accurately nonrespondents could be identified using their previous panel participation behavior and socio-demographic variables with (1) pooled logit regression modeling, compared with (2) random-effect logit regression modeling. The results are presented in Fig. 3 (accuracy) and Fig. 4 (recall). For more information, see Table 5 with regression results for the complete time series in the Appendix.

The accuracy curves in Fig. 3 show the total accuracy of identification of both respondents and nonrespondents in a certain wave. We observed very little to no differences in accuracy between logit and random-effect models in predicting panel response and nonresponse over time.

The recall curves in Fig. 4 show the accuracy of identification of nonrespondents in a certain wave. Again, we observed minor differences between different logit models—random-effect models were about 1% more accurate than pooled logit models on average, but by more than 2% in only two waves.

### Prediction of nonresponse and the length of time series

We also reviewed the prediction accuracy results for random-effect models including both online panel paradata predictors and socio-demographics (as the most accurate ones on average) to determine the length of the panel participation history time series required to predict future panel participation with desirable accuracy. This was carried out to address RQ4.

The accuracy curves in Fig. 3 show that we achieve more than 87% accuracy in predicting response and nonresponse with six waves of data. It is also evident that the prediction accuracy improved further over time with more data, peaking in wave 24 (91%) and declining slightly in the remaining six waves. Wave 8 is an exception, since only about 100 panellists were invited to participate.

Predicting only nonresponse is slightly less accurate and with more variability. The recall curves in Fig. 4 show that the predictive power generally improved over time with more data, but it peaked earlier than accuracy—in wave 16 (77%). We can conclude that we can achieve good accuracy with 15 waves of online panel paradata, identifying more than 3 of 4 nonrespondents in wave 16. After wave 17, about 10% of panellists were retired due to inactivity, which means that a significant portion of the sample, for which nonresponse was easy to predict, was lost. This drop of recall can be seen in wave 18, but it again increased gradually over time and almost reached wave 16 numbers in wave 29 (76%).

### Cost–benefit analysis of prediction and post-prediction treatment

**Fig. 5:**
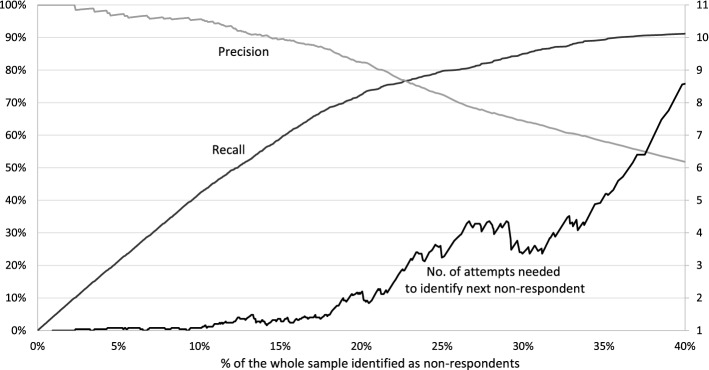
The relationship between recall and precision, “cost–benefit” analysis (wave 16, n = 2727)

To extend the findings, to turn them into practical solutions, and to answer RQ5 (cost–benefit problem), we will show the relationship between recall and precision. It will be presented conditional on the target proportion of panellists with the highest probability of nonresponse, selected to identify nonrespondents. Having in mind that organizations managing online panels could in practice identify potential nonrespondents for different purposes (e.g., see Lugtig 2014), we will show the results of our “cost–benefit” analysis. The “cost” in our case is identifying potential nonrespondents and treating them to prevent them from not participating in future panel surveys; that increases costs of panel management. The “benefit” is identifying those who would not respond in the upcoming survey(s) and successfully convincing them to participate in future panel surveys. However, as identification cannot be 100% accurate, we would also treat respondents who would normally respond without interventions.^6^ Our cost–benefit analysis is in the form of the number of attempts needed to identify the next nonrespondent by selecting the panellist with the next highest calculated probability of nonresponse (probability calculated with random-effect model, range 0–1). For this particular exercise, we used the data for the wave with the highest recall score (wave 16). The results are shown in Fig. 5.

In Figs. 1, 2, 3, 4 the selected number of panellists with the highest calculated probability of nonresponse equaled to the actual number of nonrespondents in the subsequent wave. But in Fig. 5, we are showing the relationship between precision and recall at different proportions of panellists selected for nonresponse identification. The recall and precision curves cross at about 23%, which was the nonresponse rate in wave 16. When approximately the same proportion of the whole sample are identified as nonrespondents, the recall curve starts flattening. In practice, the result of this flattening means a higher proportion of false positives. This is confirmed with the line showing the number of attempts needed to identify the next nonrespondent—while almost every panellist with the top 10% (top decile) calculated probability of nonresponse is an actual nonrespondent (1 attempt or just above 1 attempt), we would need about three attempts, including two false positives, to identify one nonrespondent with a calculated probability around the 75th percentile of probability of nonresponse. We could argue that in that region costs already exceed benefits—for example, to decrease nonresponse, we would offer extra monetary incentives to three potential nonrespondents, but only one of them would actually skip participation in that particular wave without the treatment. With our models, we could correctly identify 90% (or more) of all nonrespondents, but for a high price of about five false positives for one true positive for the last few nonrespondents to reach recall = 0.9. This chart shows how different approaches, either more or less conservative or progressive, can be taken based on expected cost–benefit balance.

To address RQ5, we showed a practical example of identification cost–benefit analysis in a particular wave. We determined that the right balance between “costs” and “benefits” when identifying nonrespondents was around the expected response rate in the upcoming wave.^7^ There are a few practical reasons for identification of nonrespondents, some of which could later become voluntary attritors. They could be treated with tailor-made incentives or special panel maintenance approaches (e.g., thank-you or birthday cards) to increase response, which could lead to better representation, higher data quality, more complete time-series, or a delayed recruitment of a refreshed sample. The other aim of identification could as well be inviting panellists, conditional on their response propensity, to achieve higher response rates while controlling for other representation errors. There might be other uses of accurate identification of less active panellists and all the above should be tested carefully and experimentally. Nonetheless, we would argue – based on the results presented in the paper—that paradata and the types of analyses we have conducted can help with the targeting of interventions.

## Discussion

Online panel paradata, which are considered a new class of paradata and are classified as the “prior survey phase” type of paradata (McClain et al. 2019), capture the entire history of panel activity for each member (Callegaro 2013). As such, they offer significant research opportunities from a methodological perspective, as illustrated in this study, and can contribute to the development and implementation of various panel management solutions. Baker et al. (2010) argued that at the very least the differences between respondents and nonrespondents should be characterized, although this is in practice seldom carried out. Moreover, the richness of this type of data might also aid understanding of panel members’ behavior, predict their future participation, and adjust panel management activities. On the one hand, the longitudinal nature of the data can have negative effects on total survey error (Groves et al. 2009), as nonparticipation bias can gradually increase over time due to differential nonresponse and voluntary attrition. On the other hand, in contrast to cross-sectional questionnaire navigation and device paradata, online panel paradata can be restructured into longitudinal panel data for inclusion in different panel data analysis models. Our results partially support the assumption that controlling for unobserved heterogeneity could improve our understanding of what predicts nonparticipation, and, even more importantly, the accuracy of regression models investigating panel participation.

In this study, we first identified some level of differential nonparticipation. The findings on the predictors of nonresponse mostly accord with the findings published by other authors such as Watson and Wooden (2009) who reported lower response rates in an Australian annual household panel survey among the youngest and the oldest participants, the least educated and those not born in Australia, but no differences in response rates by gender. Any differences in differential nonresponse between the studies could be attributed to the differences in panel types (offline household panel study vs. online panel study) and frequencies of survey data collection (annually vs. monthly). The findings related to attrition in our study were somewhat similar to those presented in the literature, which still offers contradictory evidence, and it is generally understood that demographic variables have less explanatory power than socio-psychological variables (e.g., Cheng et al. 2016; Lugtig 2014). Generally speaking, differential voluntary attrition was less severe than differential nonresponse in our study. While this may be regarded as positive from a long-term panel representation perspective, it also indicates that voluntary attrition is more challenging to predict with socio-demographic characteristics.

The variables derived from the online panel paradata, such as the survey outcome rates or the consecutive waves with a particular survey income, were shown to be reasonably good predictors of panel participation. A promising level of accuracy and consistency of prediction was achieved in identifying nonrespondents, but not voluntary attritors, by using predictors derived from online-panel paradata in pooled logit models, and random-effect models controlling for unobserved heterogeneity. While the differences were very small, we found evidence that using random-effect models (instead of pooled logit models) adds more value than including socio-demographics, in contrast to using online panel paradata derived predictors only. Moreover, it is possible that fixed-effect within-person regression models provide a better understanding of panellists’ behavior prior to nonresponse or voluntary attrition, assuming that explanatory variables are associated with the error term. However, as previously explained, the coefficients cannot be used to calculate the probabilities of the survey outcomes in contrast to random-effect models. Ultimately, the evidence presented in this study shows that the future panel participation behavior is captured best in panel participation history, but not in panellists’ socio-demographic characteristics, and that the modeling choice makes little difference.

Building on the findings presented in this article, future research should be focused on identification of other predictors of subsequent panel participation. With existing online panel paradata derived variables we could achieve sufficient accuracy in identification of nonrespondents with 15 waves of data, with accuracy slowly increasing over time. However, we believe the predictive power could be further enhanced or the models improved in such a way as to achieve the same accuracy with shorter paradata time series. Combining the online panel paradata with other types of paradata, such as questionnaire navigation and/or device paradata, and including other socio-demographic or socio-psychological covariates that have been reported in the literature as associated with nonresponse and voluntary attrition, might increase the accuracy and should be empirically tested. Combining panel data analysis and machine learning methods, i.e., performing ensemble modeling/stacking, could represent the next step in the evaluation of panel nonresponse prediction methods. Furthermore, an alternative solution for voluntary attrition worth investigating would be the use of the same data in different statistical models, which might be a better fit for survey participation outcomes with a low average rate (such as voluntary attrition).

Besides not being able to predict voluntary attrition with sufficient accuracy, one notable limitation of our study is that it was conducted in a country with a single probability-based online panel with a high frequency of data collection, i.e., monthly questionnaire completion. Therefore, we would suggest that other online panel organizations using alternative methodological approaches to recruitment and data collection, including those managing nonprobability online panels, carry out similar research to determine the value of their paradata. Lastly, as our paper highlights the significant benefit of collecting and making online paradata available for research and panel management purposes, online panel survey practice should also focus on the application of the proposed prediction methodology and evaluation of different solutions to target nonparticipation.

## Data Availability

Due to privacy restrictions and the nature of the data (data producer is a private social research company), data cannot be made available via a data repository. All other research material, including statistical software code and results, can be made available upon request for peer-review purposes.

## Appendix

See Tables 4, 5, 6

**Table 4:** Survey response percentage and attritor sample statistics (n = 2990)

	n	Survey response %		Voluntary attritor (in any wave, in %)	
		Mean	SD	No	Yes
Gender					
Female	1576	76.60	29.52	86.42	13.58
Male	1403	74.55	31.12	86.60	13.40
Education					
Bachelor or higher	1127	78.86	28.91	88.11	11.89
Certificate/diploma/trade	1062	73.59	30.83	87.01	12.99
Year 12 or equivalent	343	72.65	31.41	88.63	11.37
Year 11 or less	458	74.33	30.86	79.69	20.31
Capital city in state					
No	999	76.93	29.34	86.79	13.21
Yes	1966	75.59	30.21	86.52	13.48
Born in Australia					
No	820	72.75	31.81	86.10	13.90
Yes	2160	76.72	29.62	86.71	13.29
Only English spoken at home					
No	442	65.69	35.11	89.14	10.86
Yes	2547	77.32	29.03	86.06	13.94
Indigenous status					
No	2921	75.74	30.18	86.41	13.59
Yes	64	69.00	34.61	92.19	7.81
Other healthcare card					
No	1965	74.72	30.61	87.33	12.67
Yes	992	78.11	29.05	85.08	14.92
Carer status					
No	2400	74.37	30.97	85.58	14.42
Yes	582	81.11	26.32	90.38	9.62
Population^a^					
Offline	433	72.53	28.45	77.60	22.40
Online	2557	76.10	30.56	87.99	12.01
Age group					
18–24 years	239	58.44	35.33	93.31	6.69
25–34 years	403	67.75	33.61	91.07	8.93
35–44 years	418	71.10	32.76	89.71	10.29
45–54 years	518	75.56	29.42	87.07	12.93
55–64 years	636	79.62	28.53	85.53	14.47
65–74 years	532	84.83	23.35	82.71	17.29
75 or more years	237	82.20	22.28	75.95	24.05
Socio-economic indexes for areas					
Quartile 1	417	76.69	30.08	88.73	11.27
Quartile 2	520	76.76	29.88	85.00	15.00
Quartile 3	570	76.55	29.58	88.95	11.05
Quartile 4	635	75.28	30.67	84.88	15.12
Quartile 5	822	75.50	29.59	86.25	13.75
Age, mean with SD	2949			50.31 (17.18)	56.89 (16.62)
Survey response %, mean with SD	2990			78.65 (29.61)	55.93 (27.07)

^a^At profile survey (before first wave for panellist)

**Table 5:** Logit regression, random-effect and fixed-effect within-person logistic regression results, the effect of previous response trends on nonresponse in certain wave, 2990 persons, waves 1–30

	Logit regression model (a pooled model)				Random-effect within-person logistic regression model				Fixed-effect within-person logistic regression model			
	Coef	L 95% CI	U 95% CI	p value	Coef	L 95% CI	U 95% CI	p value	Coef	L 95% CI	U 95% CI	p value
Participation rate	− 2.73	− 3.40	− 2.06	< 0.001**	− 3.34	− 4.13	− 2.55	< 0.001**	− 4.98	− 5.85	− 4.10	< 0.001**
Non-contact rate	0.49	− 0.17	1.15	0.145	− 0.18	− 0.99	0.62	0.657	− 3.84	− 4.75	− 2.92	< 0.001**
Refusal rate	0.49	− 0.37	1.34	0.264	− 0.15	− 1.23	0.92	0.781	− 3.10	− 4.36	− 1.85	< 0.001**
Non-refusal rate	1.05	0.37	1.73	0.003**	− 0.11	− 0.95	0.74	0.807	− 4.45	− 5.41	− 3.50	< 0.001**
Charity rate	0.40	0.30	0.49	< 0.001**	0.68	0.53	0.83	< 0.001**	0.50	0.19	0.80	0.002**
Consecutive participation	0.03	0.01	0.05	< 0.001**	0.04	0.02	0.05	< 0.001**	0.00	− 0.02	0.02	0.962
Consecutive response	− 0.12	− 0.13	− 0.11	< 0.001**	− 0.09	− 0.10	− 0.07	< 0.001**	0.02	0.01	0.04	0.001**
Consecutive non-contact	0.55	0.52	0.58	< 0.001**	0.45	0.41	0.48	< 0.001**	0.49	0.45	0.52	< 0.001**
Consecutive refusal	1.10	0.87	1.32	< 0.001**	0.98	0.74	1.23	< 0.001**	1.02	0.77	1.27	< 0.001**
Consecutive non-refusal	0.33	0.27	0.39	< 0.001**	0.21	0.15	0.28	< 0.001**	0.32	0.26	0.39	< 0.001**
Consecutive charity donations	0.01	0.00	0.03	0.024*	0.02	0.00	0.03	0.015*	0.03	0.01	0.05	< 0.001**
Change from interview to other	0.09	0.01	0.17	0.028*	0.15	0.06	0.23	0.001**	0.24	0.15	0.32	< 0.001**
Change from other to refusal	0.51	0.20	0.82	0.001**	0.45	0.12	0.78	0.008**	0.43	0.09	0.76	0.012*
Constant	1.27	0.65	1.90	< 0.001**								
Pseudo R-squared	0.415											

Pooled logit regression and random-effect models include the following controls: gender, age, education, capital, born in Australia, only English spoken at home, Indigenous status, another health card, carer status, online/offline population and SEIFA

*Coef* model regression coefficient, *L 95% CI* lower limit of the 95% confidence interval, *U 95% CI* upper limit of 95% confidence interval

*Significant at the 0.05 level

**Significant at the 0.01 level

**Table 6:** Logit regression, random-effect and fixed-effect within-person logistic regression results, online and offline samples, the effect of previous response trends on voluntary panel attrition in certain wave, 2990 persons, waves 1–30

	Logit regression model (a pooled model)				Random-effect within-person logistic regression model				Fixed-effect within-person logistic regression model			
	Coef	L 95% CI	U 95% CI	p value	Coef	L 95% CI	U 95% CI	p value	Coef	L 95% CI	U 95% CI	p value
Participation rate	− 2.37	− 4.94	0.21	0.072	− 2.60	− 5.26	0.06	0.056	− 19.87	− 24.57	− 15.17	< 0.001**
Non-contact rate	− 1.32	− 3.84	1.21	0.307	− 1.45	− 4.03	1.12	0.269	− 14.27	− 18.95	− 9.60	< 0.001**
Refusal rate	1.14	− 1.39	3.67	0.377	1.17	− 1.40	3.75	0.371	− 9.62	− 15.30	− 3.93	0.001**
Non-refusal rate	− 0.32	− 2.88	2.23	0.805	− 0.46	− 3.07	2.14	0.728	− 15.65	− 20.49	− 10.80	< 0.001**
Charity rate	0.91	0.50	1.31	< 0.001**	0.94	0.51	1.36	< 0.001**	− 0.97	− 2.84	0.90	0.311
Consecutive participation	− 0.01	− 0.09	0.06	0.714	− 0.01	− 0.09	0.07	0.843	0.35	0.22	0.47	< 0.001**
Consecutive response	− 0.14	− 0.20	− 0.08	< 0.001**	− 0.14	− 0.20	− 0.08	< 0.001**	− 0.12	− 0.21	− 0.02	0.021*
Consecutive non-contact	0.05	0.00	0.09	0.059	0.05	0.00	0.10	0.052	0.66	0.50	0.81	< 0.001**
Consecutive refusal	0.81	0.60	1.03	< 0.001**	0.84	0.61	1.08	< 0.001**	1.03	0.65	1.41	< 0.001**
Consecutive non-refusal	− 0.01	− 0.22	0.19	0.905	− 0.01	− 0.22	0.21	0.956	0.49	0.19	0.79	0.001**
Consecutive charity donations	0.06	0.00	0.12	0.044*	0.06	0.00	0.12	0.046*	0.20	0.10	0.30	< 0.001**
Change from interview to other	0.14	− 0.24	0.53	0.462	0.16	− 0.23	0.55	0.416	0.88	0.40	1.35	< 0.001**
Change from other to refusal	1.07	0.67	1.46	< 0.001**	1.03	0.61	1.44	< 0.001**	0.88	0.36	1.40	0.001**
Constant	− 4.64	− 7.06	− 2.22	< 0.001**	− 4.65	− 7.09	− 2.21	< 0.001**				
Pseudo R-squared	0.147											

Pooled logit regression model includes the following controls: gender, age, education, capital, born in Australia, only English spoken at home, Indigenous status, another health card, carer status, online/offline population and SEIFA

*Coef* model regression coefficient, *L 95% CI* lower limit of the 95% confidence interval, *U 95% CI* upper limit of 95% confidence interval

*Significant at the 0.05 level

**Significant at the 0.01 level

## Footnotes

1 In this study, nonresponse is defined as questionnaire/single-panel-wave non-completion, and (voluntary) attrition is defined as panellists opting-out/voluntarily leaving the panel.

2 For definitions of survey outcomes see The American Association for Public Opinion Research (2016).

3 We also considered using probit models (static and dynamic), but we did not observe non-constant error variances, and the results would have been fairly similar.

4 For example, if a panellist completed questionnaires in waves 1–3, was not invited in wave 4 and refused to participate in wave 5, the participation rate before wave 6 was 0.6 or 60% (3 waves out of 5).

5 For sensitivity analysis purposes, we also compared the accuracy of (1) random-effect models with socio-demographics and (2) random-effect models without socio-demographics. That analysis supported the findings based on the pooled logit regression results presented above.

6 Treatment could be any panel management solution proven to increase survey completion of less frequent respondents.

7 It could be estimated by reviewing panel survey completion trends over time.

## Code availability

Code can be made available upon request. Please contact the corresponding author.
